# JAK3 Staining and CD68+ Macrophage Counts Are Increased in Patients with IgA Nephropathy

**DOI:** 10.3390/diagnostics16030437

**Published:** 2026-02-01

**Authors:** Mateus Justi Luvizotto, Precil Diego Miranda de Menezes Neves, Cristiane Bitencourt Dias, Lecticia Barbosa Jorge, Luis Yu, Luísa Menezes-Silva, Magaiver Andrade-Silva, Renato C. Monteiro, Niels Olsen Saraiva Câmara, Viktoria Woronik

**Affiliations:** 1 Department of Nephrology, Faculty of Medicine, University of São Paulo, São Paulo 00458-020, Brazilluisyu@usp.br (L.Y.); viktoriaw@usp.br (V.W.); 2 Laboratory of Transplantation Immunobiology, Institute of Biomedical Sciences, University of São Paulo, São Paulo 05508-000, Brazil; 3 Centre de Recherche sur l’Inflammation, Institut National de la Santé et de la Recherche Médicale and Centre National de la Recherche Scientifique (INSERM and CNRS), Université Paris Cité, 75018 Paris, France

**Keywords:** IgA nephropathy, JAK/STAT pathway, CD68+ macrophages, renal histopathology

## Abstract

**Background/Objectives**: IgA nephropathy (IgAN) is the most common primary glomerulopathy worldwide; it is characterized by a complex pathophysiology involving several inflammatory pathways. The Janus kinase/signal transducer and activator of transcription (JAK/STAT) pathway may be critical in this process. This study aimed to investigate the role of this pathway in IgAN and examine related tissue inflammatory markers. **Methods**: We analyzed 63 biopsy-confirmed patients with IgAN and performed immunohistochemical analysis on renal samples. A panel of antibodies targeting the JAK/STAT pathway, including JAK2, JAK3, p-STAT, STAT3, and MAPK/ERK, was used for this analysis. Six kidney tumor border samples were used as controls. Additionally, CD68 staining was used to evaluate tissue inflammation in the kidney biopsies. **Results**: Patients with IgAN showed a significantly higher cellular density of JAK3 staining at the glomerular level compared to controls, indicating JAK3 activation (*p* < 0.0002). Nevertheless, the correlation between JAK3 positivity in glomeruli and clinical parameters such as the initial and final estimated glomerular filtration rate (eGFR) and proteinuria was not statistically significant. Identical results were obtained with CD68+ macrophage counts in the glomerular compartment, which did not show any correlation with clinical parameters, while CD68+ tubulointerstitial staining demonstrated a significant correlation with both initial (*p* = 0.002) and final eGFRs (*p* = 0.0014), proteinuria (*p* = 0.010), and interstitial fibrosis (*p* < 0.001), as well as with renal disease progression (*p* = 0.005). **Conclusions**: Activation of the JAK/STAT pathway was observed in patients with IgAN relative to controls, notwithstanding the inability to assess the full pathway due to technical limitations. Macrophage CD68 staining in the tubulointerstitial area increased and was associated with clinical and laboratory parameters such as eGFR and proteinuria. Additionally, MEST-C histological parameters, such as segmental glomerulosclerosis (S0/S1), tubular atrophy/interstitial fibrosis (T0/T1/T2), and crescents (C0/C1/C2), were associated with a higher number of CD68+ cells.

## 1. Introduction

IgA nephropathy (IgAN) is the most common primary glomerular disease worldwide and a frequent cause of chronic kidney disease [1]. In the VALIGA study, which followed 1130 patients over a 7-year period, nearly one-quarter of the participants progressed to chronic kidney disease, highlighting the long-term risk of renal function decline in this population [2]. The pathophysiology of IgAN has not yet been fully established; in addition to the multi-hit mechanism, several inflammatory pathways may be involved in the disease [3,4]. Various molecules have been linked to the activation of inflammatory pathways during cytokine signaling, with the Janus kinase/signal transducer and activator of transcription (JAK/STAT) pathway being one of the most studied, as it plays a key role in the recognition of pro-inflammatory factors by various cell subtypes. This pathway is responsible for intracellular signaling and signal transduction and is involved in various biological processes, including immune system regulation, cell differentiation and proliferation, and apoptosis [5,6]. The JAK family has four members, including JAK1, JAK2, JAK3, and receptor tyrosine kinase 2 (TYK2), as well as seven STATs, and JAK/STAT signaling regulates more than 50 cytokines and growth factors [7]. In chronic kidney disease, all members of the JAK/STAT signaling pathway have been described in human kidney tissue biopsies, especially in inflammatory diseases such as lupus nephritis, IgAN, and diabetes [8,9,10,11]. Shen CL et al. showed higher JAK/STAT pathway activation in pediatric patients with focal segmental glomerulosclerosis, with STAT3 levels predicting disease severity [12]. Recent studies have demonstrated that the JAK/STAT pathway functions as a key indicator of signaling activity and it is involved in the progression of chronic kidney disease, notably promoting diabetic kidney disease by regulating autophagy in podocytes [13]. Inhibitors of the JAK/STAT signaling pathway have been explored as a potential therapeutic strategy in diabetic kidney disease [14]. In addition, IgAN patients’ peripheral blood monocytes showed upregulated STAT production after cytokine stimulation [9]. Yamada et al. demonstrated that the JAK2/STAT1 signaling pathway is involved in the enhanced production of galactose-deficient IgA1 (Gd-IgA1) in IgA patients; this process is mediated by leukemia inhibitory factor (LIF), a member of the IL-6 cytokine family. In addition, JAK2 inhibition can block the aberrant O-glycosylation pathway [15].

In animal models of lupus nephritis (NZB/WF1 rats), inhibition of the JAK/STAT pathway slowed down the progression of renal inflammation and significantly reduced IgG deposition, T-cell and macrophage infiltration, and inflammatory cytokine levels [16]. In clinical settings, some investigators have shown that JAK/STAT upregulation in podocytes, tubular-epithelial cells, and mesangial cells is associated with the progression of diabetic nephropathy [17], while treatment with an oral JAK1/2 inhibitor in diabetic mice diminished the pathological changes caused by JAK2 overexpression [18].

Recent studies have indicated that macrophages and the mononuclear cell system play crucial roles in IgAN progression. Macrophage infiltration is linked to the release of pro-inflammatory cytokines and chemokines, which are recognized as important factors in the pathogenesis of kidney disease [19]. However, the relationships between macrophage numbers/invasion sites and the mechanisms, clinical manifestations, and prognosis of IgAN remain unclear [20]. Macrophages infiltrating different kidney compartments have been correlated with different clinical-pathological features in IgAN patients: macrophage infiltration in the glomeruli has been correlated with the severity of hematuria and crescent formation, while macrophages in the tubulointerstitial compartment are linked to increased proteinuria and interstitial lesions [21,22]. In histopathological IgA settings, Kawasaki et al. [23] described an association between glomerular macrophages and the subclasses M1 and C1 of the MEST-C classification, while Soares et al. [24] associated them with subclass E1. In addition to glomerular infiltration, CD68+ tubulointerstitial macrophage infiltration is often observed in patients with IgAN; however, it has a weak correlation with the likelihood of response to immunosuppressive treatment, while it is strongly associated with lower estimated glomerular filtration rates (eGFRs), proteinuria, and poorer renal outcomes [22]. Considering that there are unresolved questions regarding inflammatory pathways in IgAN patients in the current literature, our study aimed to explore the involvement of the JAK/STAT signaling pathway and macrophage CD68 expression in kidney tissue, as well as their possible interplay with clinical and histological parameters. To the best of our knowledge, studies exploring the interplay between JAK/STAT signaling and macrophage-driven inflammation in IgAN progression are lacking.

## 2. Materials and Methods

### 2.1. Participants

A retrospective analysis was conducted on all patients diagnosed with IgAN via kidney biopsy between 2002 and 2022 at the Nephrology Department of the Hospital das Clínicas, University of São Paulo School of Medicine, São Paulo, Brazil. Clinical and laboratory data were collected at baseline and at the end of the follow-up period. Progressor patients were defined as those who progressed to end-stage renal disease (ESRD). Remission was defined as proteinuria less than 0.5 g/day and a ≤25% reduction in eGFR. The glomerular filtration rate was estimated based on the CKD-EPI. Reference ranges for analyzed biochemical parameters are: serum creatinine (0.6–1.2 mg/dL), proteinuria (<0.15 g/24 h), serum IgA levels (69–382 mg/dL), and complement C3 levels (90–180 mg/dL;). Anemia was defined as a hemoglobin level < 12 g/dL for females and <13 g/dL for males.

### 2.2. Inclusion and Exclusion Criteria

We included all patients aged 18 years or older who were diagnosed with IgAN based on a renal biopsy during the study period. Patients with incomplete medical records or missing renal biopsy data were excluded from the study.

### 2.3. Histological Study

Kidney biopsy fragments were analyzed using optical microscopy, which included the following histological stains: Masson’s trichrome, hematoxylin and eosin, methenamine silver, and periodic acid-Schiff. Six patients diagnosed with kidney tumors were submitted to nephrectomy and the marginal tissue of the tumor was used as the control for immunohistochemistry studies.

Immunofluorescence microscopy was performed using antibodies against immunoglobulin G, M, and A; complement 3 and 1q; kappa and lambda light chains; and fibrinogen. The intensity of deposition was quantified as +, ++, or +++. The Oxford Classification (MEST-C) is a standardized histopathologic scoring system for IgAN that incorporates mesangial hypercellularity (M1, defined as ≥50% of glomeruli demonstrating mesangial hypercellularity), endocapillary hypercellularity (E1), segmental glomerulosclerosis/adhesion (S1), tubular atrophy/interstitial fibrosis (T1: 26–50%; T2: >50%), and cellular/fibrocellular crescents (C1: crescents in <25% of glomeruli; C2: crescents in ≥25%). This classification has been validated as an independent prognostic tool. In the present cohort, the complete MEST-C scoring system was applied to all renal biopsy samples [25,26].

### 2.4. Immunohistochemistry

Formalin-fixed, paraffin-embedded (FFPE) tissue sections were cut with a thickness of 2 μm. Antigen retrieval was performed using citrate buffer (pH 6.0). Paraffin blocks were incubated in a drying oven at 60 °C for 30 min prior to staining. Primary antibodies included anti-JAK3 (Santa Cruz Biotechnology, Dallas, TX, USA), anti-STAT3 (Abcam, Cambridge, UK), anti-pSTAT3 (Abcam, Cambridge, UK), anti-JAK2 (Cell Signaling Technology, Danvers, MA, USA), and anti-MAPK/ERK (Cell Signaling Technology, Danvers, MA, USA). CD68 (Santa Cruz Biotechnology, Dallas, TX, USA) was employed as a marker for M0 macrophages, and detection was carried out using streptavidin conjugated to horseradish peroxidase (SPB-125; Spring, Pleasanton, CA, USA), followed by development with Stable DAB (Spring). Immunohistochemical staining for pSTAT3, STAT3, and JAK2 in both glomerular and tubulointerstitial compartments did not yield specific or interpretable results; the observed signals were indistinct from nonspecific diffuse background staining. So far, technical issues have limited our study to only partial activation of the JAK/STAT pathway.

### 2.5. Image Analysis

QuPath software, Edinburgh, UK, version v0.6.0, was used to quantify the glomerular cells positive for JAK/STAT pathway markers. Histological sections were evaluated under a microscope, and images of the glomeruli were captured at 400× magnification. The number of positive cells in the glomerular images was counted and normalized to the glomerular area to calculate cell density. From these measurements, the average count was extracted for statistical analysis to ensure the accuracy and reliability of the obtained data. Tubulointerstitial JAK/STAT expression was semi-quantified over the total area and graded as the fraction of stained cell area as follows: (1) <25% of stained cells, (2) >25% weakly stained cells, (3) >25% strongly stained cells. For the analysis of the CD68 marker, cell quantification was performed for both the glomerular and tubulointerstitial compartments. The final count was expressed as cells per glomerulus and cells per field.

### 2.6. Statistical Analysis

Statistical and graphical analyses were performed using R software (R Foundation for Statistical Computing, Vienna, Austria), version 2022.07.2 and GraphPadSoftware, Inc., San Diego, CA, USA, version 10.0. The Shapiro–Wilk test was used to assess data normality. Parametric data are presented as means ± standard deviation, while non-parametric data are expressed as medians with interquartile ranges (IQRs). For hypothesis testing, the chi-square test (with continuity correction) was used for categorical variables. Comparative analyses of numerical data between the two groups were conducted using the unpaired *t*-test or the Mann–Whitney test, as appropriate, based on the distribution of the data. Renal replacement therapy (RRT)-free survival in men and women was analyzed using Kaplan–Meier curves. Correlations were assessed using Pearson’s correlation test for parametric variables and Spearman’s correlation test for non-parametric variables. Statistical significance was set at *p* < 0.05.

## 3. Results

Table 1 presents the clinical, biochemical, and histopathological characteristics of patients with IgAN. Among the 63 patients included in the analysis, 35 (55.6%) were female, and 46 (73.0%) self-identified as white. The median age was 33 years, with an interquartile range (IQR) of 24.5 to 46.0 years. Proteinuria (≥1 g/day) or a protein/creatinine ratio ≥ 1 g/g was observed in 82.5% of the patients. The median serum creatinine level was 1.39 mg/dL (range: 0.9–2.2 mg/dL), corresponding to an eGFR of 58 mL/min/1.73 m^2^ (range: 31–95 mL/min/1.73 m^2^), as calculated using the CKD-EPI formula.

The median serum albumin concentration was 3.5 g/dL (range: 3.1–3.7 g/dL), and hematologic parameters showed a mean hemoglobin level of 12.8 g/dL (±1.8), while the mean serum IgA level was 368.9 mg/dL (±155.3) and the mean serum C3 concentration was 125.8 mg/dL (±40.7), resulting in an IgA/C3 ratio of 2.6.

The MEST-C score, which indicates mesangial hypercellularity, endocapillary hypercellularity, segmental sclerosis, tubular atrophy/interstitial fibrosis, and the presence of crescents, was applied to all biopsy specimens. Mesangial hypercellularity (M1) was observed in 76.2% of patients, while segmental sclerosis (S1) was present in 79% and endocapillary hypercellularity (E1) was identified in 38.1% of cases. Tubular atrophy and interstitial fibrosis were detected in 33.3% of patients, with 19% classified as T1 and 14.3% as T2. Crescents were observed in 28.5% of cases, of which 23.8% were categorized as C1 and 4.7% as C2.

Patients were followed up for a mean duration of 8.33 ± 5.3 years, with only 2 out of 63 patients lost the follow-up. At the end of the follow-up period, the mean eGFR was 44.0 mL/min/1.73 m^2^, ranging from 13.0 to 86.5 mL/min/1.73 m^2^, with a median annual decline of 1.19 mL/min/1.73 m^2^ (range: −3.95 to −0.19 mL/min/1.73 m^2^). Hypertension was present in 46.6% of the cohort. In terms of disease progression, only 28.5% of patients achieved remission, while 25.8% progressed to end-stage renal disease.

Considering the association between MEST-C histopathological classes and eGFR at the time of biopsy, S1 patients showed lower eGFRs than S0 patients {53.5 (31.0–89.7) vs. 103.0 (43.0–112.0) *p* = 0.034}, as did T1/T2 patients compared to T0 patients {31.0 (25.0–40.0) vs. 83.0 (49.0–105.5) *p* < 0.001}, while the lower values in E1 patients were only borderline statistically significant {45.5 (26.7–63.2) vs. 83.0 (38.5–107.5) *p* = 0.05}. In addition, considering the final eGFR, E1 patients exhibited lower values {12.5 (8.7–49.2) vs. 65.0 (34.0–92.0) *p* = 0.014}, as did S1 {38.5 (9.0–81.7) vs. 70.0 (40.0–101.0) *p* = 0.031} and T1/T2 {12.0 (6.6–30.0) vs. 61.0 (34.5–92.5) *p* < 0.001} patients.

However, no significant differences in initial or final eGFR were found between patients with and without crescents (C1/C2 compared to C0), nor between those with and without mesangial hypercellularity (M1/M0), as shown in Table 2.

During follow-up, around 30% of patients developed ESRD, with data from only two patients missing. A comparative analysis of the clinical parameters between patients who progressed to ESRD and those who did not on conclusion of the study, as shown in Table 3, revealed that progressor patients were younger; more frequently had hypertension, higher serum creatinine, and lower eGFR and plasma C3 levels, with persistent hematuria; and had a greater eGFR decline during follow-up, indicating a shorter time to develop ESRD. Regarding histological data, it was observed that patients who progressed to ESRD had a higher degree of segmental sclerosis and tubulointerstitial involvement compared to those who did not progress.

### 3.1. Immunohistochemical Staining for JAK3 and MAPK/ERK

**Figure 1:**
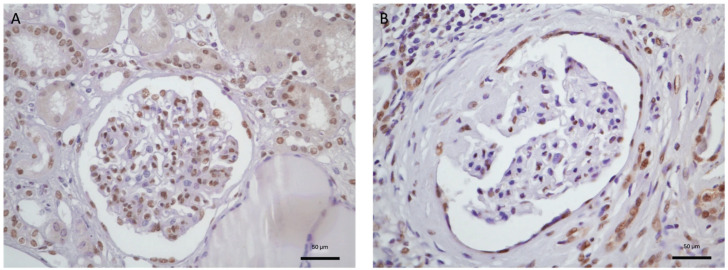
Immunohistochemical staining images of JAK3 in a patient with IgAN (**A**) and in a control subject (**B**) (×400 magnification).

Unstained renal biopsy slides from 63 patients diagnosed with IgAN were examined for the expression of JAK3 and were compared with controls (Figure 1).

**Figure 2:**
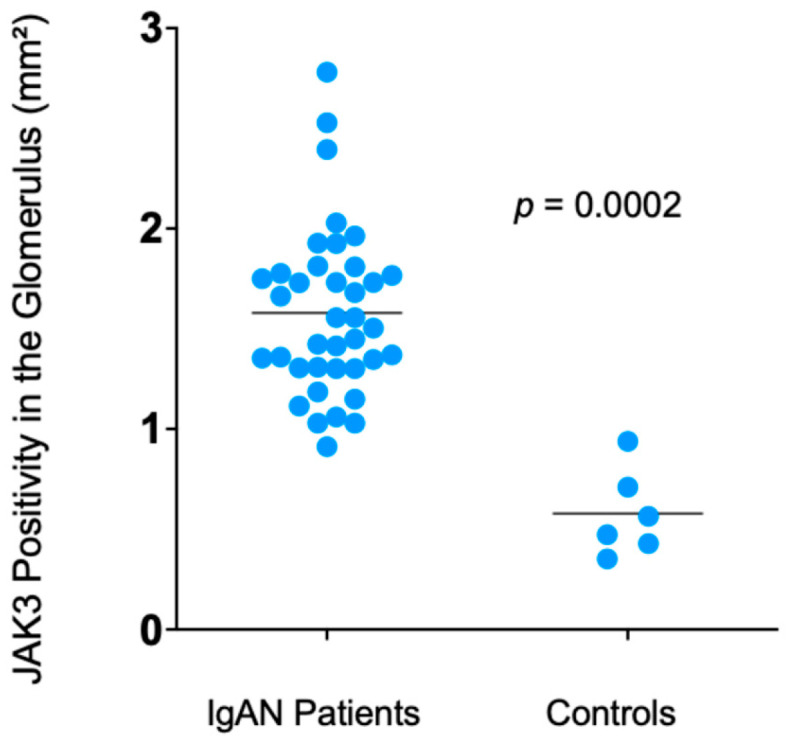
Glomerular JAK3 count differences between IgAN patients and controls.

Regarding the JAK/STAT signaling pathway, a higher JAK3 glomerular cell density was observed in patients with IgAN than in controls {1.55 (1.32–1.79) vs. 0.51 (0.43–0.67) *p* = 0.0002} (Figure 2). Meanwhile, tubulointerstitial JAK3 expression was only borderline significantly increased over controls {2.0 (2.0–3.0) vs. 1.5 (1.0–2.0) *p* = 0.045}.

**Figure 3:**
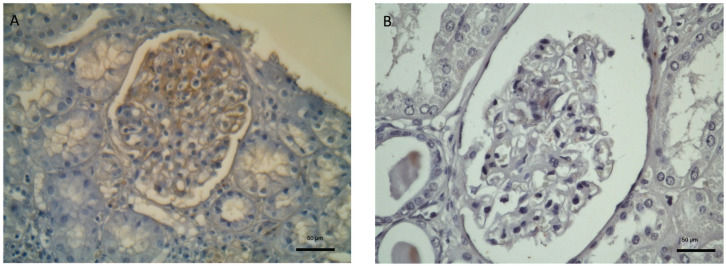
Immunohistochemical staining images of MAPK/ERK in a patient with IgAN (**A**) and a control subject (**B**) (×400 magnification).

Immunohistochemical staining for the Mitogen-activated protein kinase/extracellular signal-regulated kinase (MAPK/ERK) pathway showed enhanced expression in the glomerular compartment that was present in over 50% of mesangial cells, pointing to increased activity in IgAN patients compared to controls (Figure 3).

### 3.2. Immunohistochemical Staining for CD68

Considering CD68 staining, the glomerular count was 2.9 ± 2.3 cells per glomerulus, with no correlation with the evaluated clinical, laboratory, and histological parameters. In the same population, the interstitial CD68+ count was 15.9 ± 19.7 cells per field, which was significantly correlated with initial serum creatinine, initial eGFR, final eGFR, initial proteinuria, and interstitial fibrosis. These findings are summarized in Table 4.

Upon evaluating the association between interstitial CD68 positivity and the histological MEST-C score, significant correlations were observed with segmental glomerulosclerosis (weak correlation, R = 0.25, *p* = 0.047), tubular atrophy/interstitial fibrosis (strong correlation, R = 0.53, *p* < 0.0001), and the presence of crescents (moderate correlation, R = 0.39, *p* = 0.0012). In contrast, no correlation was found between glomerular CD68+ positivity and MEST-C parameters. Table 5 summarizes the correlations between CD68+ expression and the MEST-C score.

**Figure 4:**
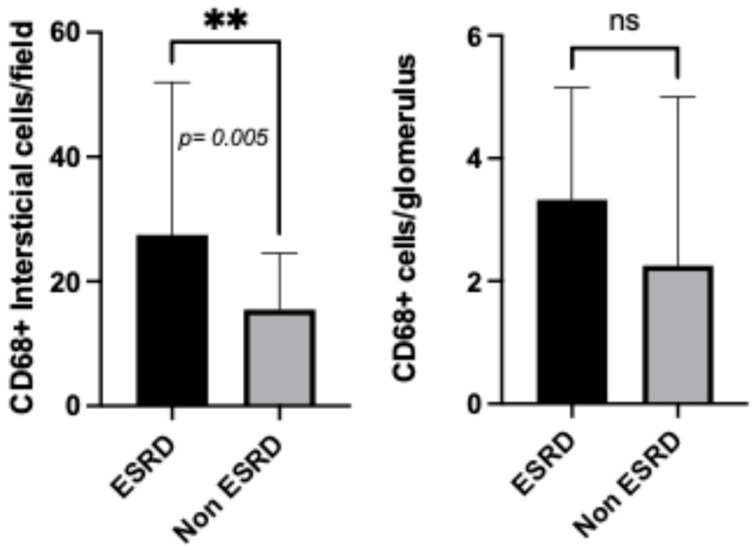
CD68+ cell counts in patients according to ESRD (end-stage renal disease). ** indicates a *p* value < 0.005.

When evaluating disease progression, patients who progressed to dialysis had higher interstitial CD68+ cell counts compared to non-ESRD patients {27.46 (15.41–49.42) vs. 15.47 (8.54–23.59) *p* = 0.0050}, as demonstrated in Figure 4. However, no significant differences in glomerular compartment cell counts were observed when comparing ESRD patients and non-ESRD patients {3.32 (1.90–5.05) vs. 2.25 (1.56–4.50) *p* = n.s.}.

## 4. Discussion

This study aimed to evaluate the inflammatory mechanisms mediated by macrophages and the JAK/STAT signaling pathway in IgAN. We observed an increased density of JAK3 staining in glomeruli compared to controls, indicating JAK3 activation. Nevertheless, we could not demonstrate any correlation between this increased expression and clinical or histological parameters, nor with remission or progression to dialysis. JAK3 has been shown to be selectively expressed in glomerular epithelial cells, indicating a potentially specific role in glomerular signaling pathways. In the context of IgAN, elevated expression levels of JAK3 in these cells have been associated with a decline in renal function. These findings further support the involvement of the JAK/STAT signaling pathway in the pathogenesis of IgAN and suggest that JAK3, in particular, may be associated with disease progression, perhaps through its effects on glomerular cell activation and injury [27]. According to the literature, unrestricted activation of the JAK/STAT pathways may contribute to mesangial cell activation in IgAN. A study comparing 70 patients with histologically confirmed IgAN to healthy individuals demonstrated that JAK signaling was more pronounced in patients with IgAN than in controls. Additionally, increased pSTAT1 and pSTAT3 activity was observed in both the glomerular and tubulointerstitial areas of the kidneys in patients with IgAN [9].

Evidence of a marked increase in p-STAT3-positive glomerular cells in patients with IgAN, relative to normal kidney tissue, suggests that STAT3 activation may be critically involved in glomerular inflammation and the pathogenesis of disease progression [28]. A previous study demonstrated that increased STAT3 expression is a key driver of elevated galactose-deficient IgA1 production. Furthermore, inhibition of this pathway, mediated by interleukin-6 using a specific STAT3 inhibitor, was shown to reduce the production of galactose-deficient IgA [29].

Previous studies have shown that MAPK/ERK signaling is activated in patients with proteinuria exceeding 1 g/day [30]. However, in our study, despite observing increased deposition of this pathway in the mesangial region, no significant correlations were found with proteinuria, renal function, or other parameters. Similarly to our findings, Faria et al. did not observe a significant association between proteinuria or eGFR and increased p-ERK1/2 expression [31]. MAPK/ERK activity was shown to be present in the glomeruli and tubulointerstitial regions in another study involving patients with various glomerulopathies. However, patients with IgAN were not included in that study, which limits direct comparison with our data [32].

Regarding tissue inflammatory mechanisms, where CD68+ macrophages play an important role, we observed significant associations with the laboratory and histological parameters of IgAN patients, such as significant correlations between interstitial CD68 positivity and both eGFR and proteinuria. However, no such correlations were observed with glomerular CD68 expression. These results suggest that interstitial macrophage infiltration, as indicated by CD68 expression, may be more closely associated with the progression of renal dysfunction in patients with IgAN, whereas glomerular macrophages may not have the same relationship with these clinical parameters, and may only be related to acute glomerular inflammation. To further support this statement, we demonstrated through regression that dialysis as an endpoint and persistent hematuria were associated with interstitial CD68 expression (*p* = 0.004 and *p* = 0.02, respectively), but not with glomerular CD68 expression; however, these associations lost significance after adjustment in the multivariable analysis. Furthermore, our results are in accordance with previous published studies that demonstrated increased CD68 expression in the tubulointerstitial compartment, showing positive correlations with serum creatinine levels and proteinuria [33].

When correlating histological parameters with the MEST-C score, our data showed that interstitial CD68 expression was correlated with interstitial fibrosis, sclerosis, and crescents, while glomerular staining did not show any correlation with these parameters. In contrast, Hu et al. found a higher count of glomerular CD68+ cells in samples with M1, S1, and C1 lesions, while increased expression in tubulointerstitial areas was observed only in S1 and T1 [33]. These findings corroborate those of other studies that have assessed the role of these markers in IgAN. For instance, Caliskan et al. published a study involving 47 patients with IgAN and reported an association between macrophages and tubular atrophy, interstitial fibrosis, and proteinuria [34]. Silva et al. evaluated macrophages as predictors of a poor prognosis in IgAN and found a positive association with worse renal outcomes [21]. Soares et al. demonstrated a correlation between glomerular CD68 positivity and endocapillary hypercellularity in a pathological study, enabling pathologists to assess endocapillary hypercellularity parameters (E1) more precisely through CD68 tissue staining [24]. In summary, there are major unresolved questions in the current literature regarding CD68+ macrophage infiltration and its role in the pathogenesis and clinical outcomes of IgAN.

Given the interconnected mechanisms of tissue inflammatory activation, represented by CD68+ cell infiltration, and the potential activation of the JAK/STAT pathway, we conducted correlation studies between these pathways but found no significant associations.

This study is one of the first to evaluate the JAK/STAT signaling pathway in the context of IgAN, contributing valuable insights into the potential role of this pathway in renal injury. The findings contribute to providing a novel perspective on the involvement of immune-mediated pathways in this disease. However, despite extensive efforts to adapt and optimize our immunohistochemical technique, we observed partial activation of the JAK/STAT pathway. These technical limitations hindered our ability to fully assess the pathway’s role in IgAN, underscoring the need for further methodological refinement in future studies.

## Figures and Tables

**Table 1:** Baseline clinical and histological characteristics of patients with IgAN.

	**N = 63**
Age (years)	33.00 (24.50, 46.00)
Female sex (n/%)	35 (55.6)
Race	
White (n/%)	46 (73.0)
Non White (n/%)	13 (20.6)
East-Asian (n/%)	4 (6.3)
Serum creatinine mg/dL	1.39 (0.94, 2.21)
Proteinuria ≥ 1 g/day (n/%)	52 (82.5)
Proteinuria g/day	1.60 (1.11, 3.06)
eGFR by CKD-EPI (mL/min/1.73 m^2^)	58.0 (31.0, 95.0)
Hematuria (n/%)	54 (85.7)
Serum albumin g/dL	3.50 (3.10, 3.77)
IgA mg/dL	368.9 ± 155.3
C3 mg/dL	125.8 ± 40.7
IgA/C3	2.60 (2.26, 3.49)
Hemoglobin (g/dL)	12.80 ± 1.83
Hypertension (n/%)	39 (61.9)
Oxford Classification (n/%)	
M1	48 (76.2)
E1	24 (38.1)
S1	50 (79.4)
T1/T2	20 (33.3)
C1/C2	18 (28.5)

eGFR by CKD-EPI: estimated glomerular filtration rate using the CKD-EPI formula; M1: mesangial hypercellularity; E1: endocapillary hypercellularity; S1: segmental glomerulosclerosis; T1/T2: tubular atrophy or interstitial fibrosis; C1/C2: cellular crescent. Non-categorical data variables are expressed as means ± standard deviation or medians (IQR).

**Table 2:** Association of the initial and final estimated glomerular filtration rate (eGFR) with MEST-C classes.

Classification	Initial eGFR	*p*-Value	Final eGFR	*p*-Value
M0	91.0 (50.5–110.0)	0.100	66.0 (30.0–98.5)	0.236
M1	49.5 (31.0–90.0)		41.0 (10.5–82.5)	
E0	83.0 (38.5–107.5)	0.050	65.0 (34.0–92.0)	0.014
E1	45.5 (26.7–63.2)		22.5 (8.7–49.2)	
S0	103.0 (43.0–112.0)	0.034	70.0 (40.0–101.0)	0.031
S1	53.5 (31.0–89.7)		38.5 (9.0–81.7)	
T0	83.0 (49.0–105.5)	<0.001	61.0 (34.5–92.5)	<0.001
T1/T2	31.0 (25.0–40.0)		12.0 (6.6–30.0)	
C0	66.0 (37.0–104.0)	0.064	59.3 (16.4–87.7)	0.145
C1/C2	45.5 (29.5–62.7)		29.0 (8.0–60.0)	

Abbreviations: M0/M1: mesangial hypercellularity; E0/E1: endocapillary hypercellularity; S0/S1: segmental sclerosis/synechiae; T0/T1/T2: tubular atrophy/interstitial fibrosis; C0/C1/C2: fibrocellular crescents.

**Table 3:** Comparison of clinical characteristics of patients who progressed to ESRD and those who did not.

	**ESRD**		***p*** **-Value**
	**No (45)**	**Yes (16)**	
Age (years)	40.0 (28.0; 50.0)	27.0 (22.0; 2.5)	0.007
Female (n/%)	28 (62.2)	5 (31.2)	0.065
White (n/%)	34 (75.6)	10 (62.5)	
Non-White (n/%)	8 (17.8)	5 (31.2)	0.525
Asian (n/%)	3 (6.7)	1 (6.2)	
Hemoglobin (g/dL)	12.87 ± 1.87	12.74 ± 1.85	0.801
Creatinine (mg/dL)	1.14 (0.90; 1.54)	2.60 (1.62; 3.06)	0.001
eGFR by CKD-EPI (mL/min/1.73 m^2^)	64.0 (41.0; 94.0)	29.5 (22.0; 41.5)	0.001
ΔeGFR (mL/min/1.73 m^2^/year)	−0.62 (−1.79; 0.47)	−9.04 (6.5; 103.0)	0.001
Albumin (g/dL)	3.5 (3.0; 3.73)	3.55 (3.18; 3.80)	0.750
IgA (mg/dL)	359.1 (275.7; 423.2)	268.0 (233.0; 377.0)	0.599
C3 (mg/dL)	133.6 ± 42.1	110.0 ± 32.2	0.048
IgA/C3	2.94 (2.26; 3.50)	2.60 (1.99; 2.77)	0.666
Proteinuria (g/day)	1.42 (0.96; 2.80)	2.44 (1.35; 3.42)	0.108
Hematuria (n/%)	24 (84.4)	15 (93.8)	0.606
Hypertension (n/%)	24 (53.3)	14 (87.5)	0.034
M1 (n/%)	33 (73.3)	13 (81.2)	0.769
E1 (n/%)	16 (35.6)	8 (50.0)	0.473
S1 (n/%)	32 (71.1)	16 (100.0)	0.039
T1/T2 (n/%)	8 (17.8)	11 (68.8)	0.001
C1/C2 (n/%)	10 (23.3)	5 (33.3)	0.671
Follow-up (years)	10.64 (6.25; 12.93)	1.47 (0.55; 8.56)	0.001
Final hematuria (n/%)	12 (26.7)	12 (85.7)	0.001

ESRD: end-stage renal disease; eGFR by CKD-EPI: estimated glomerular filtration rate using the CKD-EPI formula; M1: mesangial hypercellularity; E1: endocapillary hypercellularity; S1: segmental sclerosis/adhesion; T1/T2: interstitial fibrosis/Tubular atrophy; C1/C2: cellular crescents. Data are expressed as means ± standard deviation or medians (IQR).

**Table 4:** Correlation between positive CD68 staining and clinical parameters, including histological interstitial fibrosis.

	Glomerular CD68+ Cells	*p*-Value	Interstitial CD68+ Cells	*p*-Value
Initial Serum Creatinine	r = 0.09	0.4701	r = 0.43	0.0004
Initial eGFR	r = −0.11	0.3943	r = −0.46	0.0002
Final eGFR	r = −0.16	0.2000	r = −0.39	0.0014
Initial Proteinuria	r = 0.07	0.5736	r = 0.32	0.0101
Interstitial Fibrosis	r = 0.20	0.1213	r = 0.59	<0.001

eGFR: estimated glomerular filtration rate.

**Table 5:** Correlation between tissue CD68 expression and MEST-C class.

	Glomerular CD68+ Cells	*p*-Value	Interstitial CD68+ Cells	*p*-Value
M1	r = 0.18	0.151	r = 0.21	0.088
E1	r = 0.20	0.119	r = 0.21	0.095
S1	r = 0.10	0.441	r = 0.25	0.047
T1/T2	r = 0.13	0.303	r = 0.53	<0.0001
C1/C2	r = 0.15	0.240	r = 0.39	0.0012

M1: mesangial hypercellularity; E1: endocapillary hypercellularity; S1: segmental sclerosis/adhesion; T1/T2: interstitial fibrosis/tubular atrophy; C1/C2: fibrocellular crescents.

## Data Availability

The original contributions presented in the study are included in the article, further inquiries can be directed to the corresponding author.

## Abbreviations

The following abbreviations are used in this manuscript:

CD68	Cluster of Differentiation 68
ESRD	End-stage renal disease
IgAN	IgA nephropathy
JAK/STAT	Janus kinase/signal transducer and activator of transcription
JAK2	Janus kinase 2
JAK3	Janus kinase 3
STAT3	Signal transducer and activator of transcription 3
pSTAT3	Phosphorylated signal transducer and activator of transcription 3
eGFR	Estimated glomerular filtration rate
MEST-C	Mesangial hypercellularity, endocapillary hypercellularity, segmental glomerulosclerosis, tubular atrophy/interstitial fibrosis and crescents
MAPK/ERK	Mitogen-activated protein kinase/extracellular signal-regulated kinase
M1	Mesangial hypercellularity
E1	Endocapillary hypercellularity
S1	Segmental glomerulosclerosis
T1/T2	Tubular atrophy/iintersticial fibrosis grades
C1/C2	Crescent grades
